# Effect of growing media and fertilization on sugarcane flowering under artificial photoperiod

**DOI:** 10.1371/journal.pone.0181639

**Published:** 2017-08-03

**Authors:** Anna L. Hale, Paul M. White, Charles L. Webber, James R. Todd

**Affiliations:** United States Department of Agriculture, Agricultural Research Service, Southeast Area, Sugarcane Research Unit, Houma, Louisiana, United States of America; USDA-ARS Southern Regional Research Center, UNITED STATES

## Abstract

The USDA-ARS Sugarcane Variety Development Program in Houma, LA aims to maximize the number of panicles available for crossing through artificial manipulation of the environment. In a three-year study, the effect of growing media, fertilizer treatment, and their interaction on sugarcane flowering (% of panicles emerged), and number of days to flowering (DTF) under an artificial photoperiod treatment were assessed. The commercially-available sugarcane cultivar, ‘HoCP 96–540’ was planted in 2.8-L pots and subjected to the standard local photoperiod treatment. The cultivar was planted in four growing media (RediEarth Seedling and Germination Mix, Fafard, Metro-Mix^®^902, and Metro-Mix^®^900) and subjected to three different fertilizer applications. In the control treatment, fertilizer application was stopped prior to the commencement of the photoperiod treatment as practiced in some sugarcane breeding programs. The continuous treatment consisted of an application of a 10 ml solution of a NPK three times a week between June and October. The partial treatment consisted of applications of the same NPK solution applied post-initiation between September and October. Nitrogen starvation prior to the commencement of the photoperiod treatment is generally accepted to improve flower initiation; thus the standard practice is to cease nitrogen application two weeks prior to beginning a photoperiod regime. The growing media used in this study did not have a significant effect on days to flowering or percent panicle emergence. In our study, the control fertilizer treatment showed a flowering percentage across all growing media types of 21.2% less than a continuous fertilization regime. Furthermore, a significant trend was observed between fertilization treatments and days to flowering, with the continuous treatment producing panicles, on average across growing media, four days earlier than the control treatment, and six days earlier than the partial treatment. Evidence across this three-year experiment indicates that we should consider modifying plant nutrition management as soil fertility was found to be inadequate.

## Introduction

A common challenge in sugarcane breeding programs is induction and synchronization of parental clones for crossing. While breeders have been aware of photoperiodism since 1920, and *Saccharum spontaneum* was induced to flower in 1938, floral induction of commercial sugarcane clones was not successful prior to 1954 [1–5]. Sugarcane flowering is physiologically complex involving multiple stages of development and requiring specific environmental conditions to achieve the flowering needed for breeding purposes (e.g. temperature, growing media moisture, and day length) [6, 7]. The use of managed photoperiod treatments applied in controlled environment facilities ensures increased initiation and flowering relative to that obtained under ambient field conditions.

Managed photoperiod regimes are utilized in subtropical, tropical, and temperate sugarcane growing regions such as Argentina, Australia [8, 9], Brazil, Ecuador [10], Pakistan, South Africa [11], Taiwan [12], and the United States. Research has been conducted in the West Indies as well, although managed photoperiod regimes are not routinely used in the breeding program [13]. In the subtropics, sugarcane does not flower freely, nor does it readily set seed; therefore, the use of managed photoperiod facilities are necessary to conduct an active crossing program and are required to control temperatures during induction and to induce flowering in most clones. Regardless of climate (tropical or subtropical), treatments are used to induce “shy flowering” clones, and to aid in flower synchronization [14].

In Louisiana, a temperate region, commercial sugarcane clones rarely flower naturally in the field during typical growing seasons. Thus the use of photoperiod treatments is required to induce flowering as needed to run a successful crossing operation. The first attempts at manipulating flower induction through photoperiod treatments in Louisiana were begun in 1948 and 1949 [15]. No photoperiod facilities were available at the time; however researchers determined that Grand Isle, LA (29°N; 89°W), a small island where temperatures are moderated by the Gulf of Mexico, offered the best hope at flower production. While flower induction was successful to some extent at the location, no seedlings were obtained, presumably because low temperatures affected pollen production. Through a series of trials that included failed attempts at flower induction due to borer infestation, and a failure to produce viable seed in low-temperature conditions, researchers were finally able to obtain viable seed in 1951 with proper pest control, and the construction of a heated greenhouse on Grand Isle, LA [15, 16].

The early work was expanded, and in 1953, the artificial manipulation of photoperiods began in Baton Rouge, LA. A “light tight house” (photoperiod bay) was constructed and utilized in the next set of experiments, and hours of daylight were artificially shortened by moving the plants into and out of this chamber based on the times of sunrise and sunset in Honolulu HI. This study demonstrated sugarcane flowering could be induced, and seed produced in Louisiana [5]. Eight additional photoperiod chambers were constructed in Baton Rouge, LA in 1954 [17], followed by the construction of a crossing house and four photoperiod bays in 1972 at the USDA-ARS Sugarcane Research Unit in Houma, LA [18]. The first year in the USDA’s new heated crossing and photoperiod facility resulted in the production of 175,930 viable seed [19, 20].

In 2010, the USDA-ARS Sugarcane Research Unit (SRU) expanded and updated the crossing facilities at the USDA Ardoyne Farm near Schriever, LA, about 20 km from Houma, LA (29°38’13.15”N; 90°50’26.29”W). Six new photoperiod bays were constructed as well as a crossing house with 99 isolation cubicles for making crosses among sugarcane parental clones. Photoperiod treatments, in place for decades, were adjusted to improve efficiency of seed production. These adjustments included increased plant spacing and plant container size, and crossing-cart automation. Further adjustments were related to nutrient solution supplied to plants, where liquid fertilizer was used to supplement slow-release, and additional nutrition was supplied to the panicles after they were cut and placed in the crossing house.

Low nutrient availability results in inconsistent flowering in sugarcane [20, 21]. Flowering is important for the sugarcane crop both in the field, where it affects overall yield, and in the greenhouse. Studies have been conducted in both locations to determine the effect of fertilizer on this trait. The Rhodesia Sugar Association Experiment Station treated field-grown commercial sugarcane with variable nitrogen levels and assessed the affect this had on flowering. The study found that nitrogen deficiency substantially increased flowering in the field, with urea producing somewhat more flowers than ammonium nitrate, and increased ammonium phosphate producing less [14]. However, the effect of nitrogen was not the same across all cultivars. Gosnell [13] found that increasing nitrogen suppressed flowering in a free-flowering cultivar but that this had little impact on a shy-flowering cultivar.

Early research in breeding programs found that for maximum flowering to occur, sugarcane growth must be vigorous; however, excessive nitrogen during the initiation period is inhibitory and the effects can be influenced by water availability [21–24]. A study conducted in Alexandria, Egypt showed that, across a wide range of field-grown sugarcane clones, low nitrogen levels were required to achieve full initiation and emergence of panicles [22].

Breeding programs focused on the production of sexual seed have experimented with altering various growing conditions, in addition to the photoperiod prescription, to increase flowering and seed set in parental sugarcane clones. Results similar to field studies were obtained by Brunkhorst [25] when studying flower initiation and emergence under controlled photoperiod conditions in South Africa. High leaf nitrogen levels at the beginning of the photoperiod treatment for floral initiation were negatively associated with initiation, emergence, and flowering. Furthermore, high levels of nitrogen two weeks into the initiation period seemed to delay flowering. Further investigations in South Africa demonstrated that a continuous application of nitrogen (2.13 g per pot) significantly increased flowering as well as panicle and fuzz weight in photoperiod-treated sugarcane clones; however, it significantly reduced the amount of viable pollen and viable seed per gram of fuzz. The role of other nutrients, including potassium, calcium, and magnesium, copper, boron, iron, manganese, molybdenum, and zinc need to be further investigated [26]. High leaf nitrogen is essential for high photosynthesis, and hence carbohydrate production; however, excessive nitrogen inhibits floral initiation in sugarcane. Traditionally, in managed photoperiod facilities, nitrogen nutrition prior to initiation has been restricted to invert the N:C ratio.

Berding et al. [8] compared potting media and fertilizer regimes to assess the effect of these components on flower induction. When comparing potting media, a coarse river sand produced almost twice the number of panicles as a multi-component growth substrate containing coarse sand, vesicular volcanic pebble, peanut shell, peat, rice husk, sawdust, and tea tree bark. The percent flowered clones were markedly superior as well. In addition, they found that a continuous nutrient supply throughout panicle initiation and development produced better emergence than a broken regime where nutrients were withheld beginning 30 days prior to initiation and continuing for another 15 days. Applying supplemental nitrogen to the multi-purpose fertilizer, however, resulted in decreased panicle emergence.

At the SRU, sugarcane parents are planted in RediEarth Plug and Seedling Mix (manufactured by Sungro Horticulture, Agawam, MA); however this mix is recommended for short-term growth of small plants. Other soil mixes are available that resist compaction and are intended for longer-term growth of larger plants.

The SRU’s newly-constructed crossing and photoperiod facility represents a unique environment in which to conduct sugarcane crossing. One of the many challenges is that low temperatures are frequently encountered during the crossing season. Previous studies demonstrated that low temperatures inhibit pollen production, thus reducing the ability to produce viable seed [6, 14–16, 27–30]. Under the currently employed photoperiod treatment, flowering occurs and crossing is conducted between September and December in Houma, LA. Air temperatures decline gradually as the season progresses, leading to decreased pollen fertility, thus reduced seed germination, late in the season.

The objective of this current study was to assess the effects of growing media and fertilizer treatments on flowering at this new facility. Increasing the number of flowering stalks, or decreasing the number of days to flowering could both positively impact viable seed production in the program.

## Material and methods

Louisiana’s leading sugarcane cultivar, HoCP 96–540 (which does not flower naturally in Louisiana), was clonally propagated using single nodes and planted into 7.62 cm Speeding^™^ trays in November 2012, 2013, and 2014. In February the following year (2013–2015) the established plants were transplanted into 2.83-L tree pots (10.2 cm x 35.6 cm; model TP414; Stuewe and Sons, Tangent, OR) which were placed in groups of 9 into milk crates on the crossing carts at the USDA-ARS Sugarcane Research Unit’s Ardoyne Farm crossing facility. Each plant was maintained as a single stalk, with extra tillers removed from the pots on a weekly basis. A slow-release fertilizer (Osmocote 19-5-8 NPK; Everris NA, Inc., Dublin, OH) was incorporated at planting (250 cc/pot). Plants were watered through drip irrigation multiple times per day and also received water through natural rainfall for the duration of the experiment. Watering times were adjusted throughout the season so that the plants were not over- or under-watered. They were never subjected to drought conditions.

Since most sugarcane clones do not flower naturally in Louisiana, all parental clones are subjected to artificial photoperiod treatments to induce flowering. Treatments begin yearly on 21 June, coinciding with the summer solstice. Parental genotypes are maintained on rail carts that are pushed into and out of photoperiod houses daily according to a set schedule. Photoperiod chambers are used to maintain a constant photoperiod of 12 h 40 min for 25 d (until 15 July). Following this constant day-length period, days are increased by 5 min (12 h 45 min) on July 16. Days are then shortened by 1 min per day until 15 September when they reach 11 h 42 min. Days are shortened in the morning, by leaving the plants inside the photoperiod house after the sun has risen. All plants in this study were subjected to this photoperiod regime. Plants remained outside under ambient conditions during the daytime hours, and were maintained in the photoperiod house during the dark period at temperatures above 24 C. Average minimum temperatures on the crossing carts ranged from 8 to 24 C across the three seasons; however during low temperature periods, the plants remained under heated conditions in the greenhouse.

The effect of fertilizer and growing media on percent flower induction and number of days to flowering was investigated in this study in a three by four factorial design with four replications.

### Growing media

Sixteen established plants of HoCP 96–540 were placed in each of four different potting media. Potting media included 1) RediEarth Plug and Seedling Mix (currently used for growing parental clones in the breeding program); 2) Fafard 52; 3) Metro-Mix^®^ 902; and 4) Metro-Mix^®^900 (all manufactured by Sungro Horticulture, Agawam, MA). The Metro-Mix^®^900 was not used for the 2015 study. RediEarth Plug and Seedling mix is designed for a wide variety of propagation applications, but is primarily used for growing small propagules in small containers. It is composed of fine Canadian Sphagnum peat moss, vermiculite, a starter nutrient charge (with gypsum), dolomitic limestone, and a wetting agent. In contrast, Fafard 52 is specifically formulated for large pots, and outdoor containers. It contains a high percentage of bark to limit soil compaction. Fafard 52 is composed of bark, Canadian sphagnum peat moss, perlite, vermiculute, dolomitic limestone, and a wetting agent. Metro-Mix^®^ 900 and Metro-Mix^®^902 are similar. Both contain bark, vermiculite, Canadian Sphagnum peat moss, perlite, and dolomitic limestone. The primary difference between them is that Metro-Mix^®^ 900 has a smaller bark size in the mix. Metro-Mix^®^ 900 is primarily used to pot plants in containers 10.16 cm and larger, and is a good choice for outdoor landscape installations and large potted indoor plants. Metro Mix 902 is well suited for growing indoor and outdoor bedding plants.

### Fertilizer treatments

Each of three fertilizer treatments was applied to a set of 16 pots, with each set containing four replications of each growing media. For the control treatment, no supplemental fertilizer was added beyond the initial incorporation of Osmocote. The “continuous treatment” consisted of an application of a 10 mL solution of 10-30-20 fertilizer (31 mg N, 94 mg P (as P_2_O_5_), and 63 mg K as (K_2_O) thrice weekly between June and October/November. Treatments began on 27 June, 2 June, and 1 June, and ended on 1 October, 20 October, and 6 November in 2013, 2014, and 2015, respectively. The “partial treatment” consisted of applications of the same fertilizer solution applied three times per week between September and October/November. Fertilizer treatment applications commenced on 6 September, 5 September, and 4 September, and ended on 1 October, 20 October, and 6 November, in 2013, 2014 and 2015, respectively. Thus, the plants in the control group received no supplemental nutrients, the continuously treated plants received them throughout the season, and the plants in the partial treatment received supplemental nutrients at the beginning and end of the season, but not during the flower initiation period (in the middle of the photoperiod treatment) when the plant transitions from vegetative to reproductive growth. Once it was determined that no additional stalks would flower, late-season applications were stopped.

### Measuring plant response

The date of panicle emergence for each plant was recorded so the number of days between the commencement of the photoperiod treatment and flowering (days to flowering; DTF) could be determined. Flowering was scored as binary data, with a 1 recorded for flowering stalks and a 0 for non-flowering.

### Statistical analysis

#### Flowering date

Because not all replications within all treatments produced a flower, the Julian dates were rank-transformed. The earliest flowering stalks received a rank of 1, and each subsequent flowering date was ranked with the next highest number. Ties were assigned the same rank, and experimental units not producing a flower received a rank one number higher than the last flowering rank. For example, in 2013, 44 panicles were produced on twelve days during the season, and the remaining stalks did not flower at all. These could have initiated, but not emerged; however this was not determined in this study. Thus, ranks in 2013 ranged from 1–13, with 1–12 being assigned to flowering stalks, and 13 assigned to those stalks that did not flower. This was repeated for each year in the analysis. Following rank transformation, flowering date data were analyzed using the pdiff option of the PROC MIXED procedure in SAS Version 9.2 (SAS Institute, Inc., Cary, NC, USA) at the α = 0.05 level. Fertilizer treatment, growing media, years, and interactions were considered as fixed variables, and replications nested within treatments were considered random. Additional analyses were conducted using PROC MIXED to determine significant differences between specific treatments using the pdiff option. These were broken down into separate analyses for treatments and growing media. Data for 2013 and 2014 were analyzed both with, and without the Metro-Mix^®^ 900 growing medium treatment.

#### Percent flowering

Because flowering data were binary and scored as presence (1) or absence (0) of a flower, a Fisher’s Exact Test was utilized to determine significant differences between growing media types and fertilizer treatments. The data were analyzed by year, and years were combined to determine the overall effect of fertilizer treatment and growing media for the duration of the study.

## Results

### Flowering date

Regardless of whether or not Metro-Mix^®^900 was included in the analysis, fertilizer treatment significantly affected flowering date, and there was a significant interaction between year and growing media at the α = 0.01 level (Table 1). The model was then reduced to include only fertilizer treatment and fertilizer treatment by medium interaction because the Metro-Mix^®^900 medium was removed for 2015 testing (Table 2). In this model, the ranks of flowering dates from continuously-treated stalks were significantly earlier than those receiving the partial or control fertilizer treatments, and no differences in flowering were detected between the partial and control treatments (Table 3). In addition, the reduced model showed no significant differences among growing media and fertilizer treatment x growing media interactions. The same results were obtained when Metro-Mix^®^900 was excluded from the analysis. When the non-flowering stalks were disregarded, the average DTF following photoperiod commencement for the continuous, control and partial treatments were 114, 118, and 120, respectively.

**Table 1:** The effect of fertilizer treatment, year, growing medium and their interactions on flowering date of HoCP 96–540 grown at USDA-ARS Ardoyne Farm near Houma, LA. Data were analyzed without (w/o) and with (w) Metro-Mix^®^900, since this growing medium was not included in the 2015 study.

Effect	w/o Metro-Mix^®^900		w/ Metro-Mix^®^900	
	F-Value	P-value	F-Value	P-Value
Fertilizer treatment	10.66	0.0042	9.83	0.0054
Year	2.67	0.0757	1.86	0.1616
Medium	0.45	0.6416	0.48	0.6953
Fertilizer treatment *Medium	0.45	0.7714	0.36	0.9033
Fertilizer treatment* Year	2.33	0.0639	2.82	0.0294
Year*Medium	6.67	0.0001	5.72	0.0001

**Table 2:** Reduced model showing the effect of fertilizer treatment, medium, and fertilizer treatment by media interaction flowering date ranks of HoCP 96–540 grown at USDA-ARS Ardoyne Farm near Houma, LA in 2013, 2014, and 2015.

Effect	F-Value	P-value
Fertilizer treatment	8.85	0.0075
Medium	0.89	0.4479
Fertilizer treatment *Medium	0.31	0.9319

**Table 3:** Average flowering date rank (rank) of HoCP 96–540 treated with either a one-time application of slow-release fertilizer (control), or with slow release as well as tri-weekly fertilizer application throughout (Continuous) or for part (Partial) of the photoperiod initiation and development period.

Effect	Rank
Control	7.24a
Continuous	4.81b
Partial	8.11a

### Percent flowering

The percentage of flowering stalks was analyzed using Fisher’s Exact Test across all years and treatments as well as in a pairwise manner. When analyzed across all years, fertilizer treatment significantly affected flowering when compared across all treatments, with the exception of the control verses partial (α = .05). Regardless of year, the percent flowering was not significantly different between the control verses partial treatments. In 2013, no significant differences were observed among or between any of the treatments (Table 4). Plants under the control treatment produced significantly fewer panicles than those which were continuously treated and those which received the partial treatment in the 2014 and 2015 flowering seasons. When averaged across seasons, 88.5% of the plants treated with continuous fertilization flowered, while only 67.3% and 55.5% of the plants receiving the control and partial fertilizer treatments flowered (Table 5).

**Table 4:** Fisher’s Exact Test P-values comparing the percent flowering in sugarcane plants treated with a one-time application of slow release fertilizer (Control) with plants receiving additional tri-weekly fertilizer application either throughout (Continuous), or for part (Partial) of the photoperiod initiation and development period.

	Fisher’s Exact Test—P-value			
	All Years	2013	2014	2015
**Continuous vs. Control vs Partial**	0.00078	0.3825	0.0146	0.0012
**Control vs. Continuous**	0.018	0.41	0.0434	0.0046
**Control vs. Partial**	0.2663	0.2213	0.716	1
**Partial vs. Continuous**	0.000289	0.716	0.0068	0.0013

**Table 5:** Average percent of flowered stalks in sugarcane plants treated with a one-time application of slow release fertilizer (Control), plants receiving additional tri-weekly fertilizer application either throughout (Continuous), or for part (Partial) of the photoperiod initiation and development period.

	Percent (%)			
	All	2013	2014	2015
**Treatment**				
**Control**	67	85	69	42
**Continuous**	89	69	100	100
**Partial**	56	60	62	33

Percent flowering was not affected by the growing medium when averaged across years. When broken down by year, the minimal pairwise differences were observed between flowering in 2014 between the RediEarth and Metro-Mix^®^902 (P = .0373;α = 0.05) and in 2013 between The RediEarth and Metro-Mix^®^900 (P = 0.0894; α = 0.1).

## Discussion

These results, while contrary to current practice in Houma’s breeding program, are consistent with those obtained by Brunkhorst [26] and Berding [8], who found that consistent nutrient availability, including nitrogen, enhanced flowering in parental clones, while excessive nitrogen can delay the process. The range of acceptable nitrogen levels in flowering clones has not been firmly established and requires additional experimentation.

The data from this study were analyzed by year, and results demonstrate natural variability among crossing seasons. In 2013, no significant differences were observed among fertilizer treatments for DTF. In the breeding program, percent flowering is routinely tracked from year to year across all parental clones on the photoperiod carts. Overall, 2013 was an excellent season, with 81% of stalks producing a flower when averaged across the two crossing facilities at the SRU, and 75% of stalks flowering at the new facility where the experiment was conducted. In 2014 and 2015, these numbers were much lower, with 63% and 69% flowering on average across the two facilities, and 59% and 63% at the new one, respectively. In 2013 ambient conditions were possibly more favorable for flowering, reducing the observable effects of the treatments. The maximum, minimum, and average daily temperature as well as precipitation were monitored throughout the experiment; however the differences between years between these variables did not readily account for the increased flowering during the 2013 crossing season. Other factors, such as light quality and intensity (which were not measured), may have attributed to these differences.

Previous studies demonstrated that avoiding excessive nitrogen during the initiation period can enhance flowering, but nitrogen deficiency can prevent emergence of already initiated panicles [24, 25, 31]. In the SRU’s breeding program, it is possible that the nitrogen starvation prior to photoperiod treatments has been taken to such an extreme that photosynthesis in the parental clones was impaired to such an extent to impact panicle development and emergence. The continuous fertilizer treatment resulted, on average, in significantly more flowering stalks that flowered earlier than the other treatments. While Brunkhorst [25] found that increased nitrogen following the initiation period may negatively affect pollen fertility and the number of seed per panicle, this was not assessed in this study. The number of panicles initiated but not emerged was not determined in this study.

The fertilizer used in this study contained nitrogen, phosphorous and potassium, thus the effect of potassium and phosphorous on flowering cannot be ruled out. There have been conflicting reports on the effect of phosphorous on flowering by other sugarcane breeding groups. Gosnell [14] reported that application of phosphorus reduced flowering, while Brunkhorst [25] and Van Dillewijn [32] showed that application significantly improved emergence, flowering, and the number of seeds per panicle. Further investigation by Brunkhorst [26] showed that the application of phosphorous reduced the number of flowering stalks unless it was applied in combination with a micronutrient mixture containing Ca, Mg, B, Cu, Fe, Mn, Mo, and Zn. When applied together, flowering was increased.

Potassium has also been previously shown to affect flowering. When a high level of potassium was applied, emergence and the number of viable seed per panicle were increased in one study (25); however in a follow-up study potassium decreased fuzz weight when not applied in combination with a micronutrient mixture [26]. When taking into consideration results from other researchers, it cannot be assumed that nitrogen alone is responsible for the results from the current study.

Managed photoperiodic initiation of sugarcane parental clones in temperate breeding programs is time consuming and costly, yet necessary, primarily because of temperature constraints, to ensure a steady supply of sexual seed for varietal selection. While standard practice in Louisiana is to starve the sugarcane plants of nitrogen prior to the commencement of photoperiod treatments, the current study indicates that this practice may have been taken to such an extreme that it is detrimental to the flowering process. The nutritional requirements of flowering sugarcane should be further explored. Furthermore, results from this study illustrate the importance and danger of observing “rules of thumb” when it comes to conducting research in which plant growth media and fertilizer formulations can vary widely.
